# Dynamic Encoding of Incentive Salience in the Ventral Pallidum: Dependence on the Form of the Reward Cue

**DOI:** 10.1523/ENEURO.0328-17.2018

**Published:** 2018-05-08

**Authors:** Allison M. Ahrens, Lindsay M. Ferguson, Terry E. Robinson, J. Wayne Aldridge

**Affiliations:** Department of Psychology, University of Michigan, Ann Arbor, MI 48109

**Keywords:** Goal Tracking, Motivation, Pavlovian Conditioning, Rats, Sign Tracking, Ventral Pallidum

## Abstract

Some rats are especially prone to attribute incentive salience to a cue (conditioned stimulus, CS) paired with food reward (sign-trackers, STs), but the extent they do so varies as a function of the form of the CS. Other rats respond primarily to the predictive value of a cue (goal-trackers, GTs), regardless of its form. Sign-tracking is associated with greater cue-induced activation of mesolimbic structures than goal-tracking; however, it is unclear how the form of the CS itself influences activity in neural systems involved in incentive salience attribution. Thus, our goal was to determine how different cue modalities affect neural activity in the ventral pallidum (VP), which is known to encode incentive salience attribution, as rats performed a two-CS Pavlovian conditioned approach task in which both a lever-CS and a tone-CS predicted identical food reward. The lever-CS elicited sign-tracking in some rats (STs) and goal-tracking in others (GTs), whereas the tone-CS elicited only goal-tracking in all rats. The lever-CS elicited robust changes in neural activity (sustained tonic increases or decreases in firing) throughout the VP in STs, relative to GTs. These changes were not seen when STs were exposed to the tone-CS, and in GTs there were no differences in firing between the lever-CS and tone-CS. We conclude that neural activity throughout the VP encodes incentive signals and is especially responsive when a cue is of a form that promotes the attribution of incentive salience to it, especially in predisposed individuals.

## Significance Statement

The incentive-motivational value of reward-paired cues varies as a function of the individual and the form of the cue itself. Here we examined whether neural activity in a brain region important for processing reward cues, the ventral pallidum (VP), reflected variation in the incentive-motivational value of a food cue, both between individuals and between different cue types (a tone versus a manipulable lever). Neural responses were greatest in individuals that attached incentive salience to a cue, and within individuals, the VP dynamically tracked changes in the motivation evoked by the different cue types. This suggests that the VP plays an important role in the encoding of the emotional/motivational states that enable cues to gain control over motivated behavior.

## Introduction

When environmental cues are paired with reward, they can acquire incentive value, and thus the ability to elicit powerful emotional/motivational states that can invigorate and/or instigate reward-seeking behavior (Bindra, 1978; Berridge, 2001; Cardinal et al., 2002). There is, however, considerable individual variation in the extent to which reward cues become attributed with incentive salience. If a discrete localizable cue, such as a lever, is paired with a food reward, some animals learn to approach and interact with the lever (sign-trackers, STs), whereas others approach the site of food delivery (goal-trackers, GTs; Zener, 1937; Boakes, 1977; Flagel et al., 2009). We have suggested that for both STs and GTs the cue acquires predictive value; however, a discrete lever cue acquires greater incentive value in STs than GTs (Robinson and Flagel, 2009; Saunders and Robinson, 2010; Yager and Robinson, 2010, 2013; Saunders et al., 2013; Robinson et al., 2014b; Singer et al., 2015; Ahrens et al., 2016b).

The extent to which reward cues are attributed with incentive salience also varies with the form of the conditioned stimulus (CS). Several features of a cue, such as stimulus modality, spatial arrangement, and localizability, can have a powerful influence on the form of the conditioned response (CR) it provokes (Holland, 1977; Holland et al., 2014). For example, in a Pavlovian conditioned approach task, a tone-CS has different effects than a lever-CS. First, the individual variation in approach seen with a lever-CS is not evident, in that a tone-CS elicits goal-tracking in all rats (Meyer et al., 2014; Beckmann and Chow, 2015). Second, a tone-CS acquires less incentive value than lever-CS, as auditory cues are weaker conditioned reinforcers than lever cues (Meyer et al., 2014; Beckmann and Chow, 2015; Singer et al., 2016). Finally, in STs, dopamine release that is evoked by a lever-CS is reduced and resembles that of GTs when only the auditory component of lever extension is available and the visual and tactile features of the lever are occluded (Singer et al., 2016).

The main target of the dopamine-rich ventral striatum is the ventral pallidum (VP), and the VP plays a critical role in mediating the reinforcing and incentive properties of cues and rewards (Smith et al., 2011; Tachibana and Hikosaka, 2012; Stefanik et al., 2013; Mahler et al., 2014; Chang et al., 2015). The VP also contains a “hedonic hotspot,” where the hedonic impact of natural rewards such as palatable food can be amplified (Smith and Berridge, 2005; Ho and Berridge, 2013; Castro and Berridge, 2014; Castro et al., 2015). Further, there is anatomic variation along the rostral-caudal axis of the VP, with anterior and posterior subregions having different connectivity patterns and different influences on reward-seeking behavior (Zahm, 2000; Kupchik and Kalivas, 2013; Leung and Balleine, 2013; Mahler et al., 2014; Root et al., 2015).

We have previously reported that the incentive value of a lever-CS is encoded in neural firing in the VP (Ahrens et al., 2016a), but we have not explored differences between rostral and caudal subregions of the VP with regard to the attribution of incentive salience to cues, nor how VP activity is influenced by the form of the CS. Therefore, we here addressed two questions. (1) Is the form of a CS (lever-CS vs. tone-CS) reflected by neural activity in the VP, when both CSs have predictive value but vary in incentive value? To do this we used a 2-CS Pavlovian conditioned approach task, in which the same rat was trained using both lever- and tone-CSs. (2) Does the neural coding of the predictive and incentive value of CSs vary based on location within the VP? We report that the VP encodes incentive value along the entire rostral-caudal axis, and the strongest cue responses occurred when STs interacted with the lever-CS. There were, however, differences in the direction of responses, with inhibition dominating in the anterior VP and excitation prominent in the posterior VP.

## Materials and Methods

### Animals

This experiment used 19 male Sprague-Dawley rats weighing 300–400 g at the start of the experiment (Charles River; Colony P04). Animals were housed in standard polycarbonate cages with pine bedding, food and water available *ad libitum*, and a reverse 12:12-h light/dark cycle. Animals were pair-housed before surgery and singly housed after surgery and were handled daily during the week before the experiment started. All procedures were approved by the University of Michigan Committee on the Use and Care of Animals.

### Surgery

Rats were anesthetized with isoflurane (1.5%–3.5%), and an incision was made on the scalp to expose the skull. Holes were drilled to allow implantation of electrode microdrives bilaterally above the VP (AP: 0.8 to –0.8; ML: 1.5–3). Some rats had one target per hemisphere (two total) some had two targets per hemisphere (four total). Electrode wire bundles were initially implanted 0.3–1 mm above the VP (DV: 6.6–7.3) and were advanced to the top of the VP (DV: 7.6) on the first day of neural recordings. Electrodes were secured with bone screws and dental cement. Rats were treated with penicillin (0.1 ml, s.c.) and flunixin (2.5 mg/kg, i.p.) for 2 d after surgery and were allowed to recover from surgery for 1 wk before the start of neural recordings.

### Apparatus

All testing took place in conditioning chambers (30.5 × 24.1 × 21 cm; Med Associates) with a modified open top enclosed in a sound-attenuating cabinet. In the center of the front wall, there was a food magazine that extended 3 cm into the chamber (Med Associates). A single retractable lever (Coulbourn Instruments) was installed to the side of the magazine. The lever was modified such that it was powered by a pneumatic air-compression system; however, the tactile features and response properties of the lever were largely unchanged from the manufacturer’s version. The chamber also contained a tone generator installed on the front wall with the lever and magazine (on the side of the magazine opposite the lever), a red house light installed on the back wall, and a white noise generator in the back of the cabinet outside the chamber (all components from Med Associates). Custom software (Mtask, Aldridge laboratory) controlled the equipment and recorded lever presses and magazine entries. The pellet dispenser was mounted 24 inches above the chamber, and pellets took ∼1 s to reach the food cup after being released from the food dispenser. Four video cameras were placed around the chamber: two aimed at the lever to record lever interaction, one behind the food cup to record magazine interaction and pellet retrieval, and one at the top of the back wall to provide a view of the entire chamber.

### Behavioral procedures

For 2 d before the start of training, rats were give 25 banana pellets in their home cages to acclimate them to this food (45 mg banana-flavored pellets; BioServ, #F0059). Rats learned to retrieve pellets from the magazine on a single pretraining day, in which 25 pellets were delivered on a variable time (VT-30 s) schedule without the lever or tone. After pretraining, rats had 10 d of training using a Pavlovian conditioned approach (PCA) procedure. In all sessions, rats were first placed in the chamber for a 1-min habituation period, then the red house light turned on and remained on for the rest of the session. Each session had 40 trials, 20 lever-CS trials and 20 tone-CS trials (see Beckmann and Chow, 2015), that occurred in pseudorandom order with no more than three of each trial type in a row. Trials were separated by VT-90 (30–150-s) intervals. On lever trials, the CS consisted of insertion of the lever into the chamber (and the illumination of the LED behind the lever) for 8 s. After 8 s, the lever retracted and a pellet was dispensed into the food cup. On tone trials, the CS consisted of a tone that played for 8 s, and when the tone turned off a pellet was dispensed. A white noise generator was on throughout the session, except it was turned off during the 8-s period when the tone was playing. PCA sessions were always identical, throughout the 10 PCA training sessions before surgery and the 8–11 PCA sessions (i.e., retraining and recording sessions) after surgery.

### Quantification of PCA behavior

Lever-CS trials produced a mix of sign- and goal-tracking behavior, whereas tone-CS trials produced only goal-tracking. Therefore, on lever-CS trials the probability of lever or magazine approach was used to determine whether rats showed ST or GT conditioned responses and to classify rats as STs or GTs. Probability was quantified as the number of trials with lever presses or food cup entry, divided by the total number of trials. Probability difference scores were calculated for each session, defined as the lever probability minus magazine probability, which resulted in scores ranging from +1 to –1. Positive scores indicated a preference for sign tracking, and negative scores indicated a preference for goal tracking. For each rat, probability difference scores were averaged across all sessions in which neural activity was recorded. Rats were classified as STs if they had scores of ≥0.4 and GTs if they had scores of –0.4 or less. Intermediate rats (those with probability difference scores between –0.4 and 0.4) were not used in the main analyses of this study.

### Video rating

All sessions were videorecorded for detailed analyses of lever and magazine interaction events. We marked the beginning and end of lever interaction within trials, defined as the interval between the first contact with the lever and the moment the rat either moved away from the lever or the lever retracted. Magazine interaction was defined as the interval between the moment the rat first put their nose into the magazine and the moment they either moved away from the magazine or the lever began to retract (i.e., the end of the CS phase). These lever and magazine interaction periods were used in the analysis of neural activity related to movement in sessions with mixed sign- and goal-tracking behavior, but were not used in the main analyses presented in the results.

### Electrodes and neural recording procedures

Custom-made 32-channel electrodes were used, one version with two recording sites and one version with four recording sites. Five rats had two recording sites targeting the same region in both hemispheres (either both anterior or both posterior). In the remaining 12 rats, anterior and posterior regions of the VP were recorded within the same animal, with four recording sites targeting both the anterior and posterior VP in both hemispheres. Electrodes had 32 10-µm nickel chromate wires (California Fine Wire Company) that were twisted into eight tetrodes of four wires each. For rats with two recording sites, wires were arranged into two bundles of four tetrodes each, and for rats with four recording sites, wires were arranged into four bundles of two tetrodes each. All electrodes had one channel designated as a reference wire, and signals were recorded with an OmniPlex D neural data acquisition system (Plexon). All electrodes were initially implanted 0.3–1 mm above the VP. Wire bundles were lowered to the top of the VP on the first recording day, and were lowered an additional 80–160 µm on subsequent days to ensure that different cells were recorded each day.

After the initial 10 d of PCA training and 1 wk of recovery from surgery, rats had 1–2 d of retraining in the PCA procedure, during which time they were tethered to the recording cable but neural data were not recorded. Only rats that resumed their previous ST/GT behavior were used in the main analyses of this study (data from a single intermediate animal were used in the analysis of motor correlates but excluded from all other analyses). After retraining, rats had 6–9 d of recording, when the wire was attached and neural data were collected throughout the duration of PCA sessions.

### Neural data analysis

Neural activity was evaluated in the intervals 12 s before CS onset to 6 s after pellet delivery. The intertrial interval (apart from the 12 s before CS onset) was excluded from analysis. Units were isolated from each other and from background noise using principle components analysis and wave form features (Offline Sorter, Plexon). Unit firing patterns were analyzed with custom software (Epochbuilder; Aldridge laboratory) and Neuroexplorer (Nex Technologies). Cross-correlations were performed to identify cells that were recorded on more than one channel, and if this occurred, only the channel with the clearest wave form shape and best unit isolation was included in analyses.

Responses to trial events were evaluated at three time points. These epochs were examined in the same manner for lever trials and tone trials. All neural activity was aligned to the moment of cue onset (time = 0 s), and all three epochs were compared to a pre-CS baseline (5 s before the onset of lever or tone cues). (1) The CS onset epoch was defined as the moment immediately after the lever extended into the cage or the tone started playing (0–0.4 s). (2) The cue exposure period was defined as the last 7 s of the 8-s period when the lever was out or the tone was playing. During this period, rats typically (but not always) engaged in sign- or goal-tracking behavior. (3) The unconditioned stimulus (US) epoch coincided with the receipt of the food pellet. The exact moment when rats retrieved the pellet with their mouth varied from trial to trial, but it typically occurred 1–2 s after the pellet was dispensed from the food dispenser. This was determined by video ratings from 20 randomly selected sessions, in which the pellet was retrieved 1.56 ± 0.07 s (mean ± SEM) after being dispensed. However, the peak of neural activity during the post-CS phase typically occurred slightly before the rats had the pellet in their mouths. Therefore, we defined the US epoch as the interval between 0.6 and 1.6 s after the pellet was dispensed, as this best captured the neural responses associated with reward delivery. For a subset of cells used in the analysis of movement-related effects, responsiveness was calculated at additional time points defined from video ratings as periods of lever interaction and magazine interaction. Average spikes/s during these intervals were compared to the 5-s precue baseline.

Any trials in which rats failed to perform a conditioned response during the cue exposure period were excluded from all analyses. For lever-CS trials, the number of trials excluded was ≤4 in 82% of sessions. Failure to perform a conditioned response was more common in tone-CS trials than lever-CS trials; however, in 68% of sessions, the number of tone trials excluded was ≤6. Sessions were required to have at least 8 nonexcluded trials to be analyzed. This resulted in 28 cells that were analyzed for responses to the lever-CS but not the tone-CS.

To determine whether a cell was responsive to trial events, we compared the firing rate (spikes/s) in each of the three epochs to the 5-s precue baseline period. We calculated *z* scores for each epoch, and cells were considered responsive if they had a *z* score ≥1.3 (excitation) or ≤–1.3 (inhibition). To examine changes in the magnitude of firing over the time course of trials, we used *z* score normalization of firing rates by dividing spikes/s in each 100–200-ms bin by the mean and standard deviation of baseline firing in the same cell.

### Histology

After the last neural recording session, we marked the terminal positions of electrode tips by passing a current through one wire on each bundle and creating a small electrolytic lesion. After 3–7 d, rats were transcardially perfused with 0.1 m phosphate buffer and 4% paraformaldehyde. Brains were sectioned coronally on a cryostat in 40-µm sections and stained for cresyl violet. Electrolytic lesions were visible with cresyl violet, and electrode placement was verified with light microscopy and comparison to a rat brain atlas (Paxinos and Watson, 2007). Only cells recorded from wire bundles that fell within the VP were included in analyses. Four rats had data from one or more bundles excluded because of missed VP placement.

### Statistics

Behavioral data from lever-CS and tone-CS trials were analyzed with linear mixed models, with CS type as a fixed factor and days as a covariate. The proportion of neurons responsive to trial events was examined with multiple pairwise χ^2^ tests comparing ST-Lever, ST-Tone, GT-Lever, and GT-Tone groups (Bonferroni corrected). Differences in the magnitude of firing during lever-CS and tone-CS trials were compared within ST and GT groups using paired *t* tests. In analyses of response magnitude, outliers were identified and removed using the ROUT method. All statistical procedures were performed with GraphPad Prism (version 7) and SPSS (version 23).

## Results

**Figure 1.**
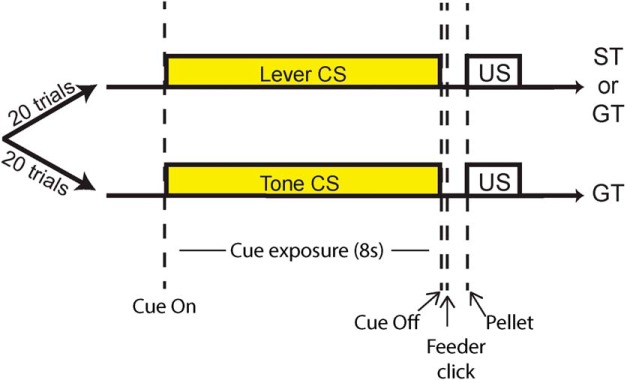
Every session had 20 lever-CS trials and 20 tone-CS trials in pseudorandom order. In lever-CS trials, a retractable lever was inserted in the cage for 8 s, then removed and followed by a banana pellet (US). Lever-CS trials elicited both sign- and goal-tracking responses, and rats were categorized as STs (*n* = 12) or GTs (*n* = 6) based on their behavior during these trials. In tone-CS trials, an 8-s tone preceded the delivery of a banana pellet. Only goal-tracking responses were observed during tone-CS trials, even in rats classified as STs.

We recorded from a total of 424 VP neurons (217 from anterior VP and 207 from posterior VP) during the performance of a two-CS Pavlovian conditioned approach task in which, on a given trial, either a lever-CS or a tone-CS predicted delivery of the same food reward (Fig. 1).

### Sign- and goal-tracking behavior during lever-CS and tone-CS trials

**Figure 2.**
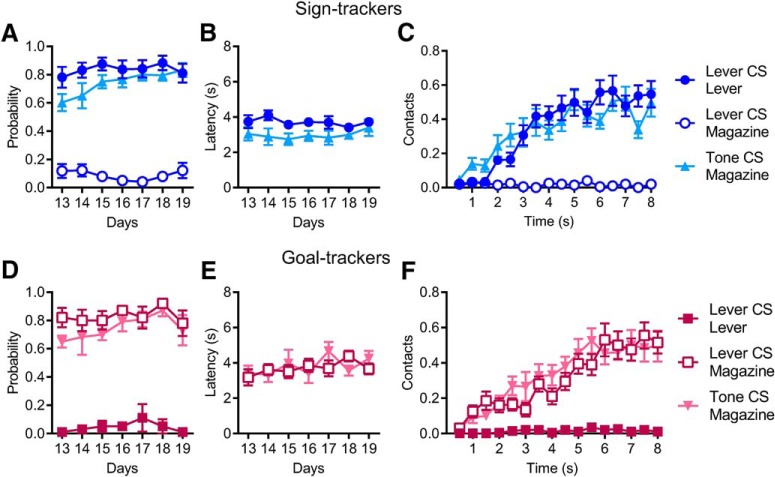
***A***, During lever-CS trials, STs showed a high probability of lever approach and a low probability of magazine approach. However, during tone-CS trials, STs showed strong goal-tracking behavior and a high probability of magazine approach that did not differ from the lever approach seen in lever-CS trials. ***B***, For STs, the latency to first contact with the lever or magazine did not differ between lever-CS and tone-CS trials. ***C***, Within trials, STs showed similar rates of contact with the lever in lever-CS trials and the magazine in tone-CS trials, both gradually increasing over the time course of the 8-s CS exposure (mean ± SEM, contacts per 0.5-s bins). ***D–F***, GTs showed a high probability of magazine approach in both lever-CS and tone-CS trials (***D***), no difference in the latency to contact the magazine between lever-CS and tone-CS trials (***E***), and no difference in the rate of contact with the magazine over the time course of lever-CS and tone-CS trials (***F***; mean ± SEM, contacts per 0.5-s bins).

The lever-CS reliably evoked a ST CR in some rats (STs, *n* = 12) and a GT CR in others (GTs, *n* = 6), whereas the tone-CS elicited a GT CR in all rats. The behavior evoked by either CS in STs and GTs are shown for sessions 13–19, when stable behavioral responses had been established and when neural activity was recorded (Fig. 2). On lever-CS trials, STs contacted the lever almost exclusively and had a very low rate of magazine contact. However, during tone-CS trials, STs approached the food magazine; i.e., made a goal-tracking CR. For STs, the probability of contacting the lever during lever-CS trials was high in all sessions. The probability of contacting the magazine during tone-CS trials began slightly lower but increased across days until it matched the probability of contacting the lever during lever-CS trials [day: *F*(1,144.2) = 10.74, *p* < 0.001; CS type: *F*(1,143.05) = 6.32, *p* < 0.05; Interaction: *F*(1,143.05) = 4.39, *p* < 0.05; Fig. 2*A*]. STs showed no significant difference in their latency to approach the lever on lever-CS trials versus the food magazine on tone-CS trials [including only the trials in which they contacted the lever or magazine; CS type: *F*(1,113.76) = 2.98, not significant (ns); Fig. 2*B*]. We also examined the rate of contact with targets of approach over the time course of 8-s CS exposure periods (i.e., lever presses and magazine entries during lever-CS trials and magazine entries during tone-CS trials, respectively) in 20 sessions (1–2 randomly chosen from each ST). For each session, average contacts were calculated in 0.5-s bins. On lever-CS trials, the rate of lever pressing increased across the 8-s trials, while magazine entries stayed very low. In tone-CS trials, rates of magazine entry were almost identical to the pattern of lever pressing seen during lever-CS trials [time: *F*(15,570) = 25.45, *p* < 0.001; CS type: *F*(1,38) = 0.04, ns; interaction: *F*(15,570) = 2.07, ns; Fig. 2*C*].

In GTs, behavior was essentially identical on lever-CS trials and tone-CS trials, as both CSs elicited a reliable GT CR. In GTs, there were no significant differences between lever-CS trials and tone-CS trials in the probability of approaching the magazine [day: *F*(1,70.04) = 2.63, ns; CS type: *F*(1,69.15) = 2.39, ns; interaction: *F*(1,69.12) =1.5, ns; Fig. 2*D*], nor in the latency to approach the magazine [CS type: *F*(1,48.9) = 0.02, ns; Fig. 2*E*]. Likewise, the rate of contacting the magazine within trials was nearly identical on lever-CS and tone-CS trials [determined from 20 sessions, 3–4 from each GT; time: *F*(15,570) = 26.2, *p* < 0.001; CS type: *F*(1,38) = 0.19, ns; interaction: *F*(15,570) = 1.4, ns; Fig. 2*F*].

### Posterior VP: sustained excitatory and inhibitory responses to a lever-CS in STs

In the posterior VP, neural responses during the period of CS exposure (i.e., the last 7 s of the 8-s interval when the lever was extended or the tone was on) were seen in all rats, but to greatly varying degrees. These responses were either sustained increases or decreases in firing which began 0.5–1 s after the onset of the CS, and either remained constant or gradually increased across the period of cue exposure.

**Figure 3.**
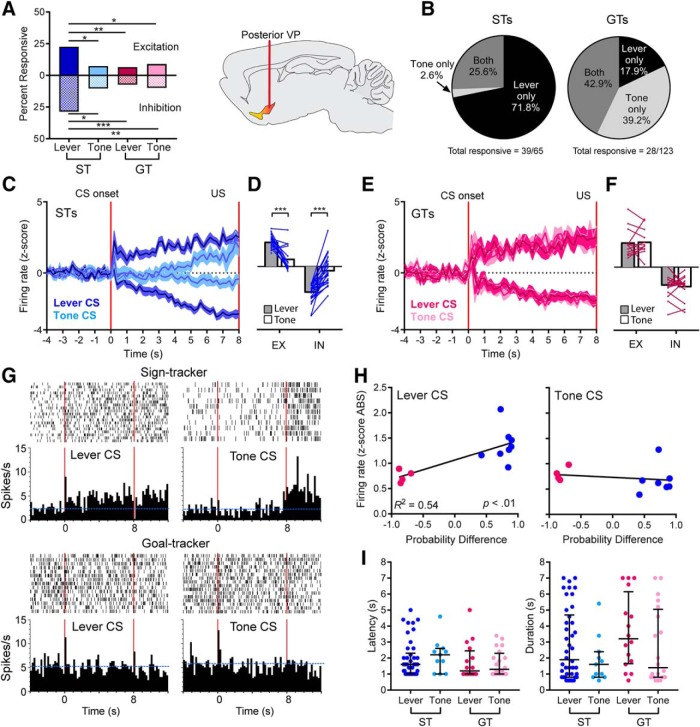
Posterior VP. Sign-tracking to the lever elicited greater neural responses than goal-tracking to either the lever or the tone. Cells were considered responsive to cue exposure if they showed a sustained increase or decrease in firing during the last 7 s of the 8-s period when the CS was present. ***A***, The percentage of cells that responded to cue exposure was significantly greater for the ST-Lever condition compared to the ST-Tone, GT-Lever, and GT-Tone conditions, and this was true for both excitatory and inhibitory responses (*, *p* < 0.05; **, *p* < 0.01; ***, *p* < 0.001). ***B***, In STs, most neurons responded only to the lever-CS and not the tone-CS; however, in GTs, responses were split more equally between the lever, the tone, or both. ***C***, In STs, the magnitude (mean ± SEM) of the cue-induced change in neural activity in responsive cells was greater during lever-CS trials than tone-CS trials. ***D***, In STs, individual responsive cells (lines) showed significantly greater increases or decreases in firing during lever-CS trials compared to tone-CS trials (***, *p* < 0.001; gray and white bars represent mean firing rate; EX, excitation; IN, inhibition). ***E***, ***F***, For GTs, the magnitude of change in responsive cells (either excitatory or inhibitory) did not differ between lever-CS and tone-CS trials (***E***) and responsive cells showed no significant differences between lever-CS and tone-CS trials (***F***). ***G***, Representative cells are shown for a ST (top) and GT (bottom). Blue horizontal dashed lines represent the baseline firing rate, and vertical red lines mark the beginning and end of the 8-s period when the cue was present. The ST cell showed greater cue-induced excitation in lever-CS trials (left) compared to tone-CS trials (right). An example GT cell showed no change during exposure to the lever or tone, although it did show a brief response to cue onset. ***H***, In lever-CS trials, probability difference scores (i.e., the tendency to sign- or goal-track) were significantly correlated with the average change in firing rate for each rat. There was no significant correlation during tone-CS trials. ***I***, No significant group differences were observed in the latency or duration of responses (figures show values for individual units, with median and interquartile ranges).

By far, the highest proportion of cue-responsive cells were found in STs on lever-CS trials, when the lever-CS evoked a ST CR. Importantly, these same cells, in the same animal and the same session, were rarely responsive on tone-CS trials, despite the fact that the tone-CS evoked a GT CR (Fig. 3*A*). In GTs, there were no differences in the number of responsive cells between lever-CS and tone-CS trials, which both evoked a GT CR, and in both conditions GTs had fewer responsive cells than STs did on lever-CS trials (Fig. 3*A*). There was a significantly greater percentage of excitatory responses for the ST-Lever condition (19/84), compared to ST-Tone (5/68; χ^2^ = 7.41, *p* < 0.05), GT-Lever (8/123; χ^2^ = 12.78, *p* < 0.01), or GT-Tone (11/123; χ^2^ = 8.66, *p* < 0.05) conditions. The same was true for the percentage of inhibitory responses, which was significantly higher for the ST-Lever condition (24/84) than the ST-Tone (7/68; χ^2^ = 7.73, *p* < 0.05), GT-Lever (9/123; χ^2^ = 16.83, *p* < 0.001), and GT-Tone (12/123; χ^2^ = 12.3, *p* < 0.01; Fig. 3*A*) conditions.

The proportion of responsive neurons in STs and GTs varied as a function of cue type. This was evaluated using only the neurons that were analyzed in both lever-CS and tone-CS conditions and were considered responsive to one or both CSs (39/65 for STs and 28/123 for GTs). In STs, most responsive neurons (28/39, 71.8%) were active only during the lever-CS condition, and some were responsive to both the lever and tone (10/39, 25.6%), but only one neuron was responsive to the tone only (1/39, 2.6%). In GTs, most responsive cells responded either to the tone alone (11/28, 39.2%) or both (12/28, 42.9%). Only 5/28 (17.9%) responded to the lever alone (Fig. 3*B*).

In STs, the magnitude of excitatory or inhibitory neural responses, in responsive neurons, also varied depending on the cue (Fig. 3*C*). In STs, the magnitude of responses during lever-CS trials was greater than on tone-CS trials, and this difference was even apparent in individual cells. Neurons that increased firing during lever-CS trials showed significantly lower firing rates during tone-CS trials [paired *t* test: *t*(14) = 5.27, *p* < 0.001]. Likewise, cells that decreased firing during lever-CS trials showed less change from baseline during tone-CS trials [paired *t* test: *t*(23) = 7.74, *p* < 0.001; Fig. 3*D*]. For GTs, in addition to having fewer responsive cells overall, the cells that were considered responsive during the CS phase showed little variation in the magnitude or time course of their response between lever-CS and tone-CS trials (Fig. 3*E*). Individual cells from GTs showed no significant differences in firing to the lever-CS versus the tone-CS, either for excitations [paired *t* test: *t*(11) = 0.22, ns] or inhibitions [paired *t* test: *t*(15) = 0.57, ns; Fig. 3*F*]. A representative ST cell shows a greater increase in firing during lever-CS trials compared to tone-CS trials (same cell in both conditions), and a representative GT cell shows no change in firing during the 8-s period of cue exposure during either lever-CS trials or tone-CS trials (Fig. 3*G*).

To address the possibility that our results may have been heavily influenced by individual rats (as some rats had more recorded cells than others), we calculated the average change in firing rates (absolute value of *z* scores) of all cells from each rat (responsive or nonresponsive). This provided a single value for each rat, which was correlated with the probability difference scores used to categorize rats as STs or GTs (STs > 0.4, GTs < –0.4). On lever-CS trials, firing rate changes were larger in STs than GTs, with a significant correlation between these rates and probability difference scores (*R^2^ =* 0.54, *p* < 0.01, *n* = 12). In contrast, average firing rates during tone-CS trials were not significantly correlated with probability difference scores (*R^2^ =* 0.04, ns, *n* = 11; Fig. 3*H*).

We also examined the latency and duration of neural responses during the cue exposure period. For each responsive neuron, we examined *z* score–normalized histograms (in 200-ms bins) and found the first instance when three or more consecutive bins crossed the threshold for responsivity (*z* scores > 1.3 or < –1.3). Latency was determined from the first bin of the response, and duration was the number of consecutive responsive bins. The first 1 s of the 8-s period of cue availability was excluded because it also captured responses to cue onset; therefore, all response latencies are reported as 1 s or longer, and the maximum duration is 7 s. There were no significant differences in the average response latency [*F*(3,85) = 0.51, ns] or response duration [*F*(3,85) = 1.44, ns; Fig. 3*I*], showing that these characteristics of cue responses did not differ between STs and GTs, or between lever-CS and tone-CS. These data suggest that group differences lie primarily in the overall population response to a cue (i.e., the proportion of responsive cells), rather than the quality or characteristics of individual unit responses.

### Anterior VP: inhibition of firing in STs dominates during lever-CS trials

**Figure 4.**
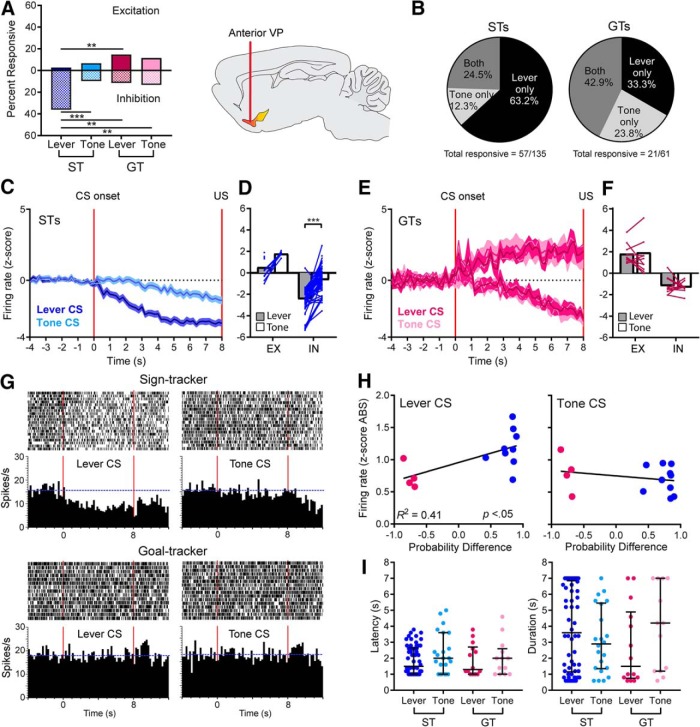
Anterior VP. STs showed sustained changes in neural activity during the cue exposure period of lever-CS trials, but these responses were predominantly inhibitory with few excitations. ***A***, The percentage of cells that showed inhibitory responses during cue exposure was significantly greater in the ST-Lever condition than the ST-Tone, GT-Lever, and GT-Tone conditions, although the percentage of excitatory responses was significantly higher for GT-Lever than ST-Lever (**, *p* < 0.01; ***, *p* < 0.001). ***B***, Most ST cells were responsive only to the lever and not the tone; however, GT cells responded to the lever only, tone only, or both at similar rates. ***C***, The average magnitude of change (mean ± SEM) is shown for inhibitions in STs, and is much stronger during lever-CS trials compared to tone-CS trials (excitatory responses were too few to graph). ***D***, STs showed significant within-cell differences in inhibition, with lever-CS trials eliciting lower firing rates than tone-CS trials (***, *p* < 0.001; gray and white bars represent mean firing rate; EX, excitation; IN, inhibition). ***E***, Among the relatively few GT cells that were responsive to cue exposure, there was no difference in the average magnitude of excitatory or inhibitory change. ***F***, Individual GT cells did not show significant changes in firing magnitude between lever-CS and tone-CS trials. ***G***, Representative cells are shown from a ST (top) and a GT (bottom). Blue horizontal dashed lines represent the baseline firing rate, and red vertical lines mark the beginning and end of the 8-s cue exposure period. The ST cell showed a reduction in firing during lever-CS trials that was not seen in tone-CS trials. The GT cell did not show significant changes in firing during exposure to the lever-CS or the tone-CS, which was typical of most GT cells. ***H***, In lever-CS trials, probability difference scores were significantly correlated with average changes in firing for each rat, though there was no correlation in tone-CS trials. ***I***, The characteristics of neural responses (latency and duration) did not differ significantly between groups (figures show values for individual units, with median and interquartile ranges).

As in the posterior VP, cells in the anterior VP showed sustained cue responses, except that responses were encoded primarily by decreases in firing during the last 7 s of the 8-s cue exposure period. In STs, many cells in the anterior portion of the VP showed a reduction in firing during exposure to the lever-CS, but not during exposure to the tone-CS. GTs did not show this inhibition during exposure to either the lever or the tone. The percentage of cells that showed inhibitory responses during the CS phase was significantly higher for the ST-Lever condition (53/147) than the ST-Tone (13/135; χ^2^ = 27.41, *p* < 0.001), GT-Lever (7/61; χ^2^ = 12.69, *p* < 0.01), and GT-Tone (8/61; χ^2^ = 10.95, *p* < 0.01; Fig. 4*A*) conditions. The percentage of excitatory responses in anterior VP was low for STs, in both lever-CS and tone-CS trials. In fact, there were significantly more excitations in the GT-Lever category (9/61) compared to the ST-Lever category (4/147; χ^2^ = 10.65, *p* < 0.01), and no significant differences compared to tone-CS categories (ST-Tone, 9/135; GT-Tone, 7/61; χ^2^ values = 0.29–6.6, ns; Fig. 4*A*).

Among the cells that were evaluated during both lever-CS and tone-CS conditions and considered responsive to one or the other (57/135 for STs and 21/61 for GTs), most ST cells were responsive during lever-CS trials alone (36/57, 63.2%), whereas 7/57 (12.3%) were responsive during tone-CS trials alone, and 14/57 (24.5%) were responsive during both trial types. For GTs, the highest proportion of cells were responsive during both the lever-CS and tone-CS trials (9/21, 42.9%), with 7/21 (33.3%) of cells responding to lever-CS trials only, and 5/21 (23.8%) responding to tone-CS trials only (Fig. 4*B*).

Firing changes were evaluated for all cells responsive during the cue exposure period, whether they were responsive to the lever only, the tone only, or both. Since only 10 cells showed excitatory responses in STs (and only 5 could be assessed during both lever-CS and tone-CS trials), this analysis is consequently focused on inhibitory responses (Fig. 4*C*). When we examined response magnitude, we found that for STs, inhibitory responses of individual cells were much stronger during lever-CS trials than tone-CS trials [paired *t* test: *t*(49) = 9.05, *p* < 0.001; Fig. 4*C*,*D*]. For GTs, neural responses during the CS phase did not differ between lever-CS or tone-CS trials, for either excitatory or inhibitory responses (Fig. 4*E*). In GTs, there were no significant differences when lever-CS and tone-CS responses were compared within each cell [paired *t* tests: excitations, *t*(9) = 0.96, ns; inhibitions, *t*(9) = 0.46, ns; Fig. 4*F*]. A representative ST cell shows greater inhibition during cue exposure in lever-CS trials compared to tone-CS trials; whereas a representative GT cell shows no response during cue exposure, during either lever-CS trials or tone-CS trials (Fig. 4*G*). From these examples, it may appear that anterior VP cells had higher baseline firing rates than posterior VP cells (compared to examples in Fig. 3*G*). However, average baseline firing did not differ between regions, with 12.03 ± 0.79 spikes/s for anterior VP (*n* = 217) and 9.7 ± 0.54 spikes/s for posterior VP (*n* = 207), and no significant difference with a nonparametric comparison (Mann–Whitney *U* = 20,709, ns). We also found that the average change in firing rates (absolute value of *z* scores) of cells from each rat were significantly correlated with probability differences scores in lever-CS trials (*R^2^ =* 0.41, *p* < 0.05, *n* = 13), but not tone-CS trials (*R^2^ =* 0.07, ns, *n* = 14; Fig. 4*H*). Finally, the latency and duration of neural responses were examined following the methods used for the posterior VP. Within responsive cells, there were no significant group differences in the latency of response onset [*F*(3,103) = 1.01, ns] or the duration of responses [*F*(3,103) = 1.1, ns; Fig. 4*I*].

**Figure 5.**
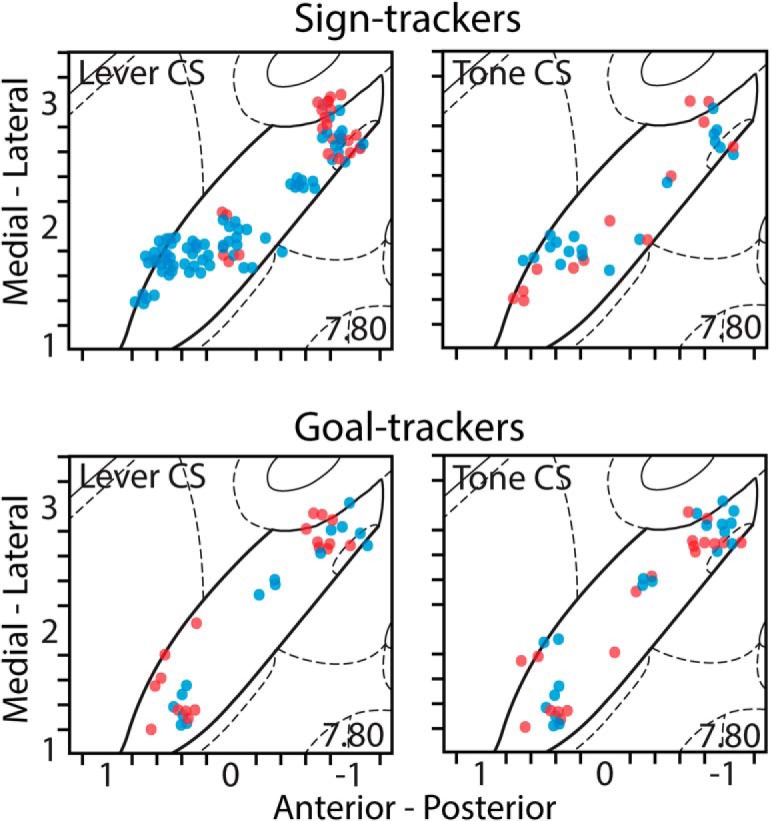
The approximate anatomic location of cells recorded in the VP that showed a sustained response during the 8-s period of cue exposure. Each dot represents a single responsive cell and indicates whether it showed an excitatory response (red) or an inhibitory response (blue). Top row, during lever-CS trials, STs showed a concentration of excitatory responses in the posterior portion of the VP and a concentration of inhibitory responses in the anterior portion of the VP, both of which are visibly diminished in the tone-CS trials. Bottom row, in GTs, both excitatory and inhibitory responses to the CS are sparsely distributed compared to the STs, with little difference between the lever-CS and tone-CS trials.

The distinction between anterior and posterior regions (AP: –0.4) was chosen based on previous research (Smith and Berridge, 2005; Smith et al., 2011); however, there is not a clear and unambiguous delineation between the two regions. Therefore, responses to the 7-s cue exposure period are shown for every responsive cell individually and mapped across the AP and ML dimensions of the VP (Fig. 5).

### STs show greater excitatory responses to CS onset on lever-CS trials compared to tone-CS trials

Data presented above focused on sustained changes in neural activity during the period of CS exposure, when rats were engaged in conditioned responding, and when the largest effects were seen. Here we focus on the more immediate response to the onset of the CS, in which differences were also observed, although they were not as large as during the rest of the CS phase. Cells in both the anterior and posterior regions of the VP showed immediate neural responses to the CSs (typically within 20–500 ms after CS onset) that were distinct and often independent from the sustained cue responses described above. The majority of cells that were responsive to CS onset (∼80%–90%) returned to baseline in <1 s. These cells may have also subsequently shown a response to the CS phase beginning ∼1–2 s (see analyses of response latency during the CS phase), but these responses were typically distinct from CS onset. In both regions, these responses were a mix of excitations and inhibitions.

**Figure 6.**
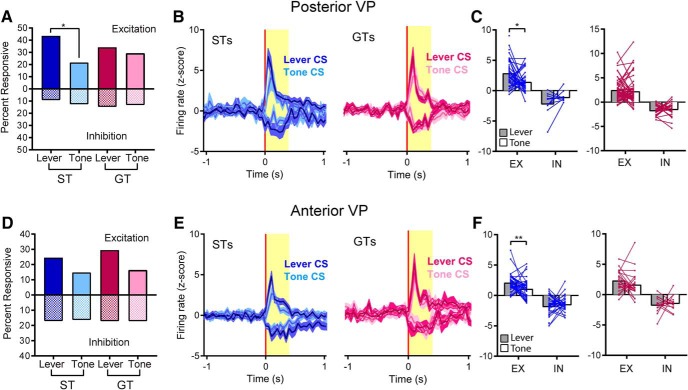
VP neurons showed immediate, phasic responses to the onset of lever and tone cues. Top figures represent cells from the posterior VP, and bottom figures represent cells from the anterior VP. ***A***, In the posterior VP, responses to cue onset were largely excitatory. The percentage of cells that were responsive to cue onset did not differ between STs and GTs, although STs had significantly more excitatory cells in lever-CS trials than tone-CS trials (*, *p* < 0.05). ***B***, Both excitatory and inhibitory responses to CS onset occurred within 0–400 ms and typically returned to baseline in <1 s. ***C***, Responses to CS onset were examined within cells in lever-CS versus tone-CS trials for STs (left) and GTs (right). STs, but not GTs, showed significantly greater excitatory firing in lever-CS trials than tone-CS trials (*, *p* < 0.05; gray and white bars represent mean firing rate; EX, excitation; IN, inhibition). ***D***, In the anterior VP, there were no significant differences in the proportion of responsive cells**. *E***, ***F***, Firing to CS onset was brief and immediate (***E***), and for STs (left), but not GTs (right), excitatory responses were significantly greater in lever-CS trials than tone-CS trials (***F***; **, *p* < 0.01).

In the posterior VP, STs had a significantly higher percentage of excitatory responses to CS onset on lever-CS trials (37/84) compared to tone-CS trials (15/68; χ^2^ = 8.07, *p* < 0.05), but otherwise there were no significant differences between groups, including when STs were compared to GTs (GT-Lever, 42/123; GT-Tone, 36/123; χ^2^ values = 0.68–4.78, ns). There were also no significant differences in the percentage of inhibitory responses (ST-Lever, 7/84; ST-Tone, 8/68; GT-Lever, 17/123; GT-Tone, 15/123; χ^2^ values = 0.01–1.47, ns; Fig. 6*A*). The basic pattern of responses to CS onset was similar in all conditions, and consisted of a brief phasic increase or decrease in firing that typically returned to near baseline levels within 500 ms (Fig. 6*B*). Individual ST cells showed greater excitation during lever-CS trials compared to tone-CS trials [paired *t* test: *t*(32) = 2.13, *p* < 0.05], although there were no within-cell differences in inhibitions, or in the magnitude of responses in GT cells (Fig. 6*C*).

In the anterior VP, the percentage of cells responsive to CS onset did not differ significantly between groups for either excitations (ST-Lever, 36/147; ST-Tone, 20/135; GT-Lever, 18/61; GT-Tone, 10/61; χ^2^ values = 0.08–5.8, ns) or inhibitions (ST-Lever, 24/147; ST-Tone, 21/135; GT-Lever, 10/61; GT-Tone, 10/61; χ^2^ values = 0–0.03, ns; Fig. 6*D*). The overall pattern of excitatory and inhibitory responses to CS onset was similar in STs and GTs, in both lever-CS trials and tone-CS trials (Fig. 6*E*), although the average magnitude of excitatory responses was significantly higher for STs during lever-CS trials than during tone-CS trials [paired *t* test: *t*(39) = 2.86, *p* < 0.01; Fig. 6*F*].

### Neural activity during the post-CS period

Neurons in both the anterior and posterior regions of the VP were also responsive throughout the post-CS period, from the time the lever was retracted or the tone turned off until the food reward (i.e., the banana pellet US) was delivered and consumed. Peak responses occurred in the interval between 0.6 and 1.6 s after the feeder click associated with activation of the pellet dispenser. We defined this period of peak neural activity “US responses,” because it encompassed the period after the feeder click signaling that the food pellet had been released from the dispenser. However, it should be noted that the exact moment of reward consumption varied from trial to trial and typically occurred slightly after this peak response; consumption of the food pellet usually occurred 1–2 s after activation of the food dispenser, as determined from video records. Therefore the US period could potentially include both anticipation of reward plus consumption of the pellet.

There were also responses to the audible click of the pellet dispenser as it released a pellet (during the first 0.4 s after the click), but these were difficult to distinguish from the sustained neural responses seen during the preceding CS phase (which ended 0.5 s before the click) and the subsequent US interval (0.6–1.6 s after the click). In both ST and GT groups, and both lever-CS and tone-CS trials, there were similar proportions of cells responsive to the pellet dispenser click (44.9% to 67.6%, with no significant differences between groups); however, most of these cells were also responsive to either the CS phase or the US (82.6% to 90.6%). Since there was not a clear and independent population-level response to this event, and no observable differences between groups, this interval was not examined further.

**Figure 7.**
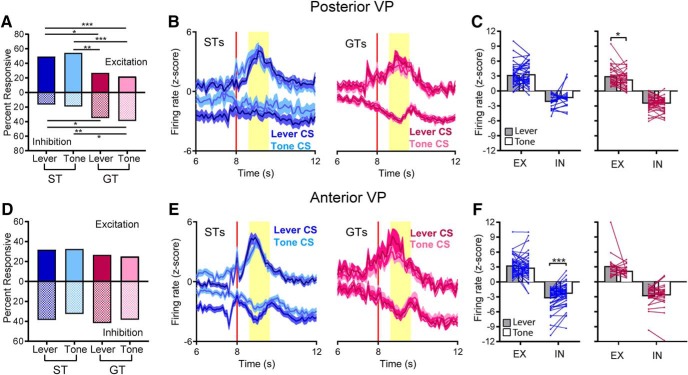
Many VP cells were responsive to presentation of the food reward (i.e., the US) in both the posterior VP (top row) and the anterior VP (bottom row). ***A***, Although there were no differences between lever-CS trials and tone-CS trials in the posterior VP, STs had a higher proportion of excitatory responses than GTs, and GTs had a higher proportion of inhibitory responses than STs (*, *p* < 0.05; **, *p* < 0.01; ***, *p* < 0.001). ***B***, Responses to the US were prominent during a 1-s period when the food pellet was delivered, which occurred 0.6–1.6 s after the CS phase ended and pellet was released from the food dispenser (shown as time = 8 s). ***C***, In STs (left) there were no significant differences in the magnitude of firing in lever-CS versus tone-CS trials, although GTs (right) showed significantly higher firing rates in lever-CS trials than tone-CS trials (*, *p* < 0.05; gray and white bars represent mean firing rate; EX, excitation; IN, inhibition). ***D***, In the anterior VP, there were no significant differences in the proportion of cells responsive to the US. ***E***, ***F***, STs (left) had significantly lower firing rates in lever-CS trials than tone-CS trials (***, *p* < 0.001), whereas GTs (right) showed no differences in firing during lever-CS versus tone-CS trials.

In the posterior VP, both STs and GTs had large proportions of cells responsive during the US period. Notably though, US-responsive neurons in STs were predominately excitatory, while GTs had predominantly inhibitory responses. There were no significant differences in percentages between lever-CS and tone-CS trials for either STs (χ^2^ values = 0.17–0.37, ns) or GTs (χ^2^ values = 0.44 – 0.79, ns). When STs were compared to GTs, in both lever-CS and tone-CS trials, the STs had more excitatory responses than GTs (ST-Lever, 34/73; ST-Tone, 35/68; GT-Lever, 36/123; GT-Tone, 27/123; χ^2^ values = 10.15–20.71, *p* < 0.05–0.001), and GTs had more inhibitory responses than STs (ST-Lever, 14/73; ST-Tone, 13/68; GT-Lever, 41/123; GT-Tone, 48/123; χ^2^ values = 5.3–11, *p* < 0.05–0.01; Fig 7*A*). The magnitude and duration of US responses were similar for both STs and GTs (Fig. 7*B*). Within individual cells of STs, there were no significant differences in the magnitude of responses during lever-CS trials versus tone-CS trials; however, for GTs, excitatory responses were significantly greater during lever-CS trials compared to tone-CS trials [paired *t* tests: *t*(36) = 2.38, *p* < 0.05; Fig. 7*C*].

In the anterior VP, there were no group differences in the proportion of US-responsive cells. The proportions of excitations did not differ significantly between STs and GTs or between lever-CS trials and tone-CS trials (ST-Lever, 40/135; ST-Tone, 39/135; GT-Lever, 15/60; GT-Tone, 12/60; χ^2^ values = 0.02–1.14, ns). Likewise, the proportions of inhibitions did not differ between groups or trial types (ST-Lever, 43/135; ST-Tone, 45/135; GT-Lever, 26/60; GT-Tone, 16/60; χ^2^ values = 0–1.5, ns; Fig. 7*D*). In STs, the magnitude of inhibitory US responses differed between trial types, with lever-CS trials eliciting a greater decrease in firing than tone-CS trials (Fig. 7*E*), although this may represent a continuation of the differences in firing seen in the previous 7-s cue epoch. For STs, individual cells were more likely to be inhibited in lever-CS trials compared to tone-CS trials [paired *t* tests: *t*(53) = 5.46, *p* < 0.001], although excitatory responses did not differ between lever-CS or tone-CS trials. Further, lever-CS and tone-CS responses did not differ among GT cells (Fig. 7*F*).

We show post-trial firing only up to 4 s after pellet delivery, and firing rates did not always return to baseline within this period. However, the trials were separated by intertrial intervals (ITIs) that ranged from 30 to 150 s, and firing reliably returned to baseline within these intervals. If it took longer than 30 s for firing to return to normal, one would expect the duration of the ITI to influence the baseline rate of the subsequent trial; however, this was not the case. Among the units that had especially pronounced firing changes during the US period (*z* score > 4), only 2 of 65 (3.1%) had significant correlations between ITIs and baseline firing in subsequent trials.

Around half of US-responsive cells also showed responses to the CS onset, the CS phase, or both. There were no significant differences in the percentages of US-responsive cells that also responded to CSs between groups (42.2%–55.3%), with the exception of posterior VP cells in ST-lever trials (91.7%, *p* < 0.001). When the ST-lever group was compared to the other groups from the posterior VP, we found a greater percentage of cells responsive to all three events (37.5% compared to 6.7%–11.8%; *p* < 0.001), or the combination of the CS phase plus the US (25% compared to GTs 5.3%–9.3%; *p* < 0.05), but there were no differences in the proportion of cells responsive to the combination of CS onset plus the US. Thus, it appears that VP cells that might normally respond to the US are also engaged by the lever-CS in STs, although this effect was only seen in the posterior VP and not the anterior VP.

### No effect of motor activity on VP firing

**Figure 8.**
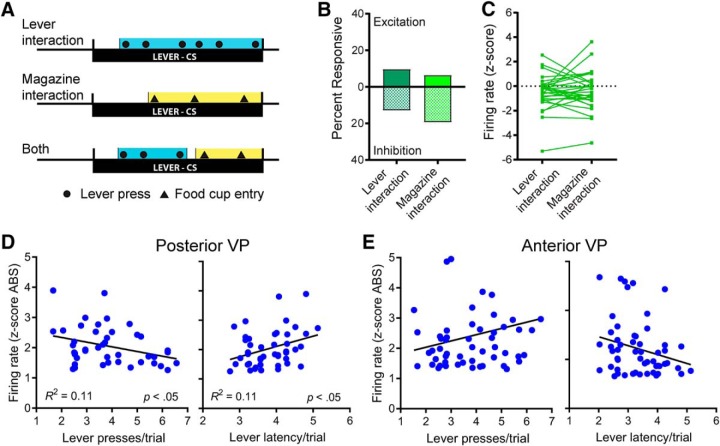
Firing in the VP was not influenced by movement patterns that differed between sign- and goal-tracking responses. ***A***, Firing rates were determined during periods of active lever interaction or magazine interaction, even if both behaviors occurred during a single trial. ***B***, ***C***, In the anterior VP, there were no differences in the percentage of cells that were responsive during lever interaction or magazine interaction (***B***), and firing rates of individual cells did not differ significantly between periods of lever interaction versus magazine interaction (***C***; see Ahrens et al., 2016a for a similar analysis of cells in the posterior VP). For STs, the strength of neural responses was not determined by the intensity of motor activity during sign-tracking as measured by lever presses per trial and latency to contact the lever. ***D***, In the posterior VP, the firing of individual neurons in STs was negatively correlated with lever presses and positively correlated with latency (*p* < 0.05), indicating slightly weaker neural responses when sign-tracking was more intense. ***E***, In the anterior VP, there were no significant correlations between neural responses and lever presses or latency.

In the results above, we found greater firing changes when rats were interacting with the lever instead of the magazine, which raises the possibility that these effects were influenced by the specific motor patterns (such as biting and pressing the lever) that systematically differed between sign-tracking and goal-tracking responses (although GTs also lick and bite the food cup; DiFeliceantonio and Berridge, 2012). In a previous study, we performed a detailed analysis of movement-related effects on firing in the posterior VP and found that differences in motor actions between STs and GTs were not responsible for differences in firing rates (Ahrens et al., 2016a). However, this previous study did not include cells from the anterior VP. Therefore, in the current study, we analyzed a subset of anterior VP cells to determine whether firing rates differed between periods of lever interaction and magazine interaction, defined as the portion of each trial in which rats were engaged in contacting the lever or magazine (Fig. 8*A*).

We analyzed 31 cells from sessions that had at least 5 trials with periods of lever interaction and 5 trials with periods of magazine interaction (tone trials were excluded). Cells from all rats (ST or GT) were combined for this analysis. Nine of these cells came from a rat classified as an intermediate, who was tested using the same methods as other rats in this study, but whose data were excluded from all other analyses. The remaining 22 cells came from STs and GTs that happened to perform their nondominant conditioned response (i.e., goal-tracking for STs and sign-tracking for GTs) in at least 5 of 20 trials. In many of these trials, rats interacted with both the lever and magazine, switching from one to the other, within the 8-s trial. Individual trials that contained both types of behavior were divided into a lever interaction portion and a magazine interaction portion that were analyzed separately.

We found that neural activity did not differ between periods of lever interaction and periods of magazine interaction. The percentage of cells that showed excitation was low, with no significant difference between lever and magazine interaction (lever interaction, 3/31; magazine interaction, 2/31; χ^2^ = 0.22, ns). While more cells showed inhibitory responses, there was again no significant difference between periods of lever interaction and magazine interaction (lever interaction, 4/31; magazine interaction, 6/31; χ^2^ = 0.48, ns; Fig. 8*B*). Furthermore, the firing of individual cells did not significantly differ between periods of lever interaction and magazine interaction trials [paired *t* tests: *t*(30) = 0.42, ns; Fig. 8*C*], showing that neural activity was the same whether rats were engaged in movements directed toward the lever or movements directed toward the magazine.

We performed an additional analysis of motor correlates using only the data recorded from STs. We observed variability in the intensity of sign-tracking responses evidenced by both the number of lever presses per trial and the latency to first contact the lever (Meyer et al., 2012). For cells responsive during the CS interaction period, we compared the change in firing rate (absolute value of *z* scores) to the simultaneously recorded behavioral measures. In the posterior VP, there was a weak but significant negative correlation between firing rates and lever presses (*R* ^2^ = 0.11, *p* < 0.05, *n* = 43) and a weak but significant positive correlation between firing rates and latency to reach the lever (*R* ^2^ = 0.11, *p* < 0.05, *n* = 45; Fig. 8*D*), both of which indicate that neural responses were less intense when sign-tracking behavior was more vigorous (although variance in one only accounts for a small percentage of variation in the other). In the anterior VP, there were no significant correlations between firing rates and lever presses (*R* ^2^ = 0.06, ns, *n* = 57) or latency (*R* ^2^ = 0.06, ns, *n* = 55; Fig. 8*E*). Altogether, these results suggest that the motor activity associated with sign-tracking is not responsible for the neural activity changes seen in STs during exposure to the lever-CS.

## Discussion

We asked how two different cues (a lever-CS vs. a tone-CS), both of which predicted delivery of an identical food reward, and both of which reliably came to produce an anticipatory CR, influenced neural firing in the VP. As expected, there was considerable individual variation in the form of the CR evoked by the lever-CS. The lever-CS evoked primarily a sign-tracking CR in some rats (STs) and a goal-tracking CR in others (GTs). However, this variation in the form of the CR was not evident on presentation of the tone-CS, which evoked a GT CR in all rats (i.e., both STs and GTs). Exposure to either CS evoked prominent neural responses during three distinct phases: (1) on initial onset of the CS, (2) during the 8-s duration of CS exposure; and (3) after CS offset and immediately before and after delivery of the food reward (US). The most dramatic changes in neural activity were observed during the 8-s period of cue exposure, when animals performed their respective CRs, but these responses varied markedly both as a function of the individual and the form of the CS. Sustained increases or decreases in neuronal firing throughout the VP were most pronounced in STs during exposure to the lever-CS, when they were actively engaged in sign-tracking behavior, relative to when the lever-CS evoked a GT CR in GTs, or when the tone-CS evoked a GT CR, in either STs or GTs. In addition, the pattern of responses differed between anterior and posterior regions of the VP, with excitatory responses prevalent in the posterior VP and inhibitory responses dominating in the anterior VP.

It is striking that there were such marked differences in neural responses evoked by two cues that were equally predictive of reward, and were equally effective in evoking a CR. Why might this be? It is well known that the form of the *US* influences the form of the CR, but it is less widely appreciated that the form of the *CS* also influences the form of the CR (Holland, 1977). We have suggested that one reason a lever-CS evokes a ST CR in some animals but a GT CR in others is because there is individual variation in the propensity to attribute incentive salience to a lever-CS. The lever-CS is an equally effective CS in STs and GTs, evoking a CR in both, but it is transformed into an attractive incentive stimulus preferentially in STs (Robinson et al., 2014b). Furthermore, a tone-CS, which evokes a GT CR in all rats, is a less effective incentive stimulus than is a lever-CS in STs (e.g., Meyer et al., 2014). Indeed, the ability of a cue to acquire incentive properties can be affected by a number of factors, such as the certainty of the reward (Anselme et al., 2013; Robinson et al., 2014a, 2015) and temporal and spatial relationships between the cue and reward (Silva et al., 1992; Burns and Domjan, 2001). Furthermore, certain combinations of visual and auditory properties cause some stimuli to become more powerful conditioned reinforcers than others (Holland et al., 2014; Meyer et al., 2014; Beckmann and Chow, 2015; Singer et al., 2016). Thus, we suggest the differences in sustained neural activity in the VP evoked by the lever-CS in STs versus GTs, and by the tone-CS, reflect the degree of incentive value attributed to these reward cues, not their predictive value (Ahrens et al., 2016a).

Indeed, during the CS phase, unit activity in the VP appeared to track dynamic changes in the incentive value of the cues, as they changed from trial to trial in a single animal. This is consistent with previous research suggesting that the magnitude of the VP response to a food-paired cue reflects the strength of that cue’s motivational impact (Tindell et al., 2005; Smith et al., 2011; Tachibana and Hikosaka, 2012; Avila and Lin, 2014; Richard et al., 2016). The most prominent firing changes were seen during the 8-s period when the cue was present (i.e., the lever was extended or the tone was playing) and corresponded with the expression of sign-tracking behavior. Presumably it is during the performance of the ST CR when the state of heightened incentive motivation is greatest. However, another possibility is that the specific motor patterns associated with sign-tracking, but not goal-tracking, could have influenced our results. In the anterior VP, we found that firing rates did not differ when STs were goal-tracking or GTs were sign-tracking within the same trial and/or session. In addition, previous studies have found that firing in the posterior VP was not affected by movements associated with sign- and goal-tracking (Ahrens et al., 2016a) or by motor activity in other reward-related tasks (Tindell et al., 2005). It is likely that the enhanced neural activity in STs, during sign-tracking, reflects the motivational state that leads to sign-tracking rather than the specific motor patterns themselves; however, we acknowledge the semi-intractable nature of motivational states and motor activity in the ST/GT model, and we cannot say with complete certainty that movement-related neural activity did not influence our results to some degree.

The VP appears to also encode the purely predictive aspects of a cue; that is, the cognitive associations that are learned during CS-US pairings, which may or may not be accompanied by a heightened incentive-motivational state (Tindell et al., 2005; Zhang et al., 2009; Smith et al., 2011). This is suggested by the immediate neural responses to CS onset in both STs and GTs and both lever-CS and tone-CS trials. In these conditions, the predictive value of the cue is constant; only the incentive value changes. Since lever and tone cues are equally effective at evoking a conditioned response in STs and GTs (meaning that both groups learn the predictive value of the cues equally well), one would not expect to see differences in prediction-related firing. Although there were no group differences between STs and GTs in response to cue onset, we did find that STs showed stronger excitatory responses to cue onset on lever trials than tone trials, which suggests an additional incentive component to these signals. In the posterior VP, we previously found a greater percentage of cells responsive to cue onset in STs than GTs (Ahrens et al., 2016a), and another recent study showed that the magnitude of immediate VP responses to a cue predicted the latency of reward seeking (Richard et al., 2016). Therefore, the immediate response to cue onset may reflect the predictive value of the cue, but it likely also carries an incentive signal to some degree, although it is less robust and consistent than firing during the more prolonged cue-exposure period, when the CRs are performed.

A high percentage of VP neurons also responded to receipt of the food reward (the US). The VP has been shown to respond to food reward even after CS-US associations are well learned and can encode the intensity of hedonic responses to palatable tastes (Tindell et al., 2006; Smith et al., 2011). In the posterior VP, US responses varied between groups (ST vs. GT) but not between trial types (lever vs. tone). This suggests that trial-by-trial changes in incentive-motivation did not have a major effect on neural responses to the US, and that instead there may be stable individual differences in the way that ST and GT rats experience the hedonic value of the US. However, in this study, we did not directly measure hedonic responses and cannot confirm whether they played a role in the current results. Furthermore, if STs and GTs experienced the reward value of the food pellet differently, they should develop conditioned behavior at different rates, which many studies have shown is not the case (Meyer et al., 2012). It should be noted that peak VP responses slightly preceded actual consumption of the pellet, and one would expect the representation of hedonic value to be time locked to US contact. It is possible that these neural responses encoded an aspect of reward anticipation rather than purely hedonic responses, although further research will be necessary to determine the exact mental states associated with these responses.

The VP is one of the primary output structures of the mesolimbic reward pathway. The VP receives prominent projections from GABAergic medium spiny neurons in the nucleus accumbens (NA), with the NA shell innervating the anterior VP and the NA core innervating the posterior VP (Heimer et al., 1991; Stefanik et al., 2013; Kupchik et al., 2015; Creed et al., 2016). However, there is also evidence for bidirectional communication between the NA and VP, as cue responses in the VP sometimes precede and drive those in the NA (Richard et al., 2016). The NA is a likely source of both the excitatory and inhibitory responses we observed in the current study. Although GABAergic inputs from the NA are inhibitory, stimulation of the NA has been shown to produce a mix of excitatory and inhibitory responses in VP neurons (Chrobak and Napier, 1993).

The different firing patterns we observed in the posterior and anterior VP (i.e., excitation versus inhibition) suggest that these regions may exert different effects on downstream structures. There are topographic differences in the projection targets of VP; for example, the ventromedial VP sends prominent projections to the mediodorsal nucleus of the thalamus and NA shell, while the dorsolateral VP projects to the substantia nigra, subthalamic nucleus, and NA core (Churchill and Kalivas 1994, 1999; Zahm et al., 1996; Leung and Balleine, 2015). In addition, neurons in both the anterior and posterior VP send projections to the ventral tegmental area (VTA), where they provide tonic inhibition of both dopamine and nondopamine neurons (Floresco et al., 2003; Creed et al., 2016). There is a great deal of evidence that dopamine signaling differs between STs and GTs (Flagel and Robinson, 2017), and patterns of dopamine release in the NA change dynamically depending on whether rats display a sign-tracking or goal-tracking CR (Singer et al., 2016). Therefore, it is plausible, although it cannot be determined from the current study, that pathways from the VP to the VTA transmit an incentive signal.

In conclusion, we report that neural activity in the VP dynamically tracks changes in the form of a CR, both within individuals and as the form of the CS varies, which we suggest reflects dynamic changes in the extent to which the CS functions as an attractive and “wanted” incentive stimulus. Indeed, the degree to which cues acquire incentive salience, as indicated by sign-tracking, is reflected in both the number of VP responsive neurons and the intensity of their responses to reward cues. It has been reported that reward cues are not very effective in engaging brain reward circuits unless they are attributed with incentive value; their predictive value is not sufficient (Flagel et al., 2011; Yager et al., 2015). The current results support this notion by showing that the VP is preferentially engaged by cues attributed with incentive salience. Thus, the VP appears to be an integral part of a brain system that generates cue-evoked emotional/motivational states (Berridge et al., 2009).

## Synthesis

Reviewing Editor: Lorna Role, Stony Brook Decisions are customarily a result of the Reviewing Editor and the peer reviewers coming together and discussing their recommendations until a consensus is reached. When revisions are invited, a fact-based synthesis statement explaining their decision and outlining what is needed to prepare a revision will be listed below. The following reviewer(s) agreed to reveal their identity: Kyle Smith, Matthew Roesch

The reviewers were fine with the revised version, as is.
